# Functional Characterization of the Intact Diaphragm in a Nebulin-Based Nemaline Myopathy (NM) Model-Effects of the Fast Skeletal Muscle Troponin Activator *tirasemtiv*

**DOI:** 10.3390/ijms20205008

**Published:** 2019-10-10

**Authors:** Eun-Jeong Lee, Justin Kolb, Darren T. Hwee, Fady I. Malik, Henk L. Granzier

**Affiliations:** 1 Department of Cellular and Molecular Medicine, University of Arizona, Tucson, AZ 85721, USA; ejlee@email.arizona.edu (E.-J.L.); justinkolb@email.arizona.edu (J.K.); 2 Research and Early Development, Cytokinetics, Inc., South San Francisco, CA 94080, USA; dhwee@cytokinetics.com (D.T.H.); Fmalik@cytokinetics.com (F.I.M.); 3 Medical Research Building, RM 325, 1656 E Mabel St, Tucson, AZ 85721, USA

**Keywords:** muscle contraction, isometric force, shortening velocity, fast skeletal muscle troponin activators, diaphragm muscle, nebulin, nemaline myopathy

## Abstract

Respiratory failure due to diaphragm dysfunction is considered a main cause of death in nemaline myopathy (NM) and we studied both isometric force and isotonic shortening of diaphragm muscle in a mouse model of nebulin-based NM (Neb cKO). A large contractile deficit was found in nebulin-deficient intact muscle that is frequency dependent, with the largest deficits at low–intermediate stimulation frequencies (e.g., a deficit of 72% at a stimulation frequency of 20 Hz). The effect of the fast skeletal muscle troponin activator (FSTA) *tirasemtiv* on force was examined. *Tirasemtiv* had a negligible effect at maximal stimulation frequencies, but greatly reduced the force deficit of the diaphragm at sub-maximal stimulation levels with an effect that was largest in Neb cKO diaphragm. As a result, the force deficit of Neb cKO diaphragm fell (from 72% to 29% at 20 Hz). Similar effects were found in in vivo experiments on the nerve-stimulated gastrocnemius muscle complex. Load-clamp experiments on diaphragm muscle showed that *tirasemtiv* increased the shortening velocity, and reduced the deficit in mechanical power by 33%. Thus, *tirasemtiv* significantly improves muscle function in a mouse model of nebulin-based nemaline myopathy.

## 1. Introduction

Nemaline myopathy (NM) causes weakness and poor tone (hypotonia) in the muscles of the face, neck, and upper limbs, and often greatly affects the respiratory muscles [1,2]. NM is a congenital neuromuscular disorder that is caused by mutations in genes that play critical roles in muscle contraction, in particular genes related to the structure and function of the thin filament [3,4]. NM mutations in the nebulin gene (*NEB*) make up a large fraction of cases [5,6], but to date many other genes have also been implicated, in particular actin alpha 1 (*ACTA1*) and in more rare cases tropomyosin 3 (*TPM3)*, tropomyosin 2 (*TPM2)*, troponin T1 (*TNNT1)*, cofilin 2 (*CFL2)*, kelch repeat and BTB domain containing 13 (*KBTBD13)*, kelch like family member 40 (*KLHL40)*, kelch like family member 41 (*KLHL41)*, leiomodin 3 (*LMOD3)*, myopalladin (*MYPN),* ryanodine receptor 3 *(RYR3),* or Myosin 18B (*MYO18B)* [7,8]. Muscle weakness in NM involves force-deficits at the sarcomere level [9], while other NM characteristics include the presence of nemaline rods (dense accumulations of proteins inside the fibers), hypotrophy, and fiber type switching [10]. Currently, no cures exist for NM. Respiratory failure is thought to be an important cause of death in NM [11,12] and, thus, insights in diaphragm contractility in NM are needed for fully elucidating the pathology of NM and for developing treatment strategies. Yet, diaphragm muscle has been less studied than peripheral muscles, due to the limited availability of diaphragm biopsies. Here we studied how the function of the diaphragm is altered in a mouse model of NM, the recently developed tissue-specific and conditional nebulin KO model, the of nebulin-based NM (Neb cKO) mouse, that phenocopies a severe form of NM [13].

Nebulin is a giant filamentous protein (~800 kDa) that is an integral component of the skeletal muscle sarcomere [14,15]. It winds around the actin filament and spans from the Z-disk to near the pointed end of the thin filament [14,16]. Nebulin is known as a thin filament stabilizer that regulates the length of the thin filament [16,17]. It plays important roles in skeletal muscle where it is involved in laterally aligning myofibrils [18] and in regulating contraction [19,20,21,22,23]. Mutations in nebulin cause alterations in thin filament structure and function that contribute to the force deficit, via reduced thin filament length [24], reduced thin filament stiffness, and altered tropomyosin and troponin behavior [25]. These changes depress force generation due to reduced thin filament activation and altered cross-bridge cycling kinetics [24,26,27,28]. Contractile weakness in respiratory muscle, relative to peripheral skeletal muscle, is considered to lead to respiratory failure, which is the main cause of death in NM patients [11]. Joureau et al. [29] have shown that contractile weakness of permeabilized diaphragm muscle fibers of a NM mouse model was more pronounced than that in soleus muscle fibers. To date, no intact muscle experiments have been performed on the NM diaphragm and, additionally, there are no effective treatment options to improve contractile weakness in NM.

As a possible therapeutic option in this work we focused on *tirasemtiv*, a small molecule that selectively activates the fast skeletal muscle troponin complex by increasing its sensitivity to calcium [30]. *Tirasemtiv* has been investigated as a potential therapy for neuromuscular disorders, such as Amyotrophic Lateral Sclerosis (ALS) and, myasthenia gravis [31,32,33,34,35]. Considering that FSTAs increase the calcium sensitivity of permeabilized muscle fibers of nebulin-deficient skeletal muscle [36], we hypothesized that *tirasemtiv* has a beneficial effect on contractile performance of electrically stimulated diaphragm muscle from the Neb cKO NM mouse model. Additionally, we investigated the effect of *tirasemtiv* on in vivo peripheral muscle strength in the NM mouse model and focused this part of our study on the gastrocnemius complex. 

We found a large specific-force deficit in the electrically stimulated diaphragm of Neb cKO muscle, relative to control muscle, that was largest at low to intermediate stimulation frequencies (~70% at 1–60 Hz). *Tirasemtiv* significantly reduced the force deficit of Neb cKO diaphragm (from ~70% to ~40%). Similar findings were made in the in vivo gastrocnemius study. Load-clamp experiments on diaphragm muscle showed that *tirasemtiv* increased the shortening velocity at all loads, which resulted in significant reduction in the power deficit of cKO. Thus, *tirasemtiv* significantly improves muscle function in a mouse model of nebulin-based NM. 

## 2. Results

### 2.1. Isometric Force Production by Diaphragm Muscle

**Figure 1:**
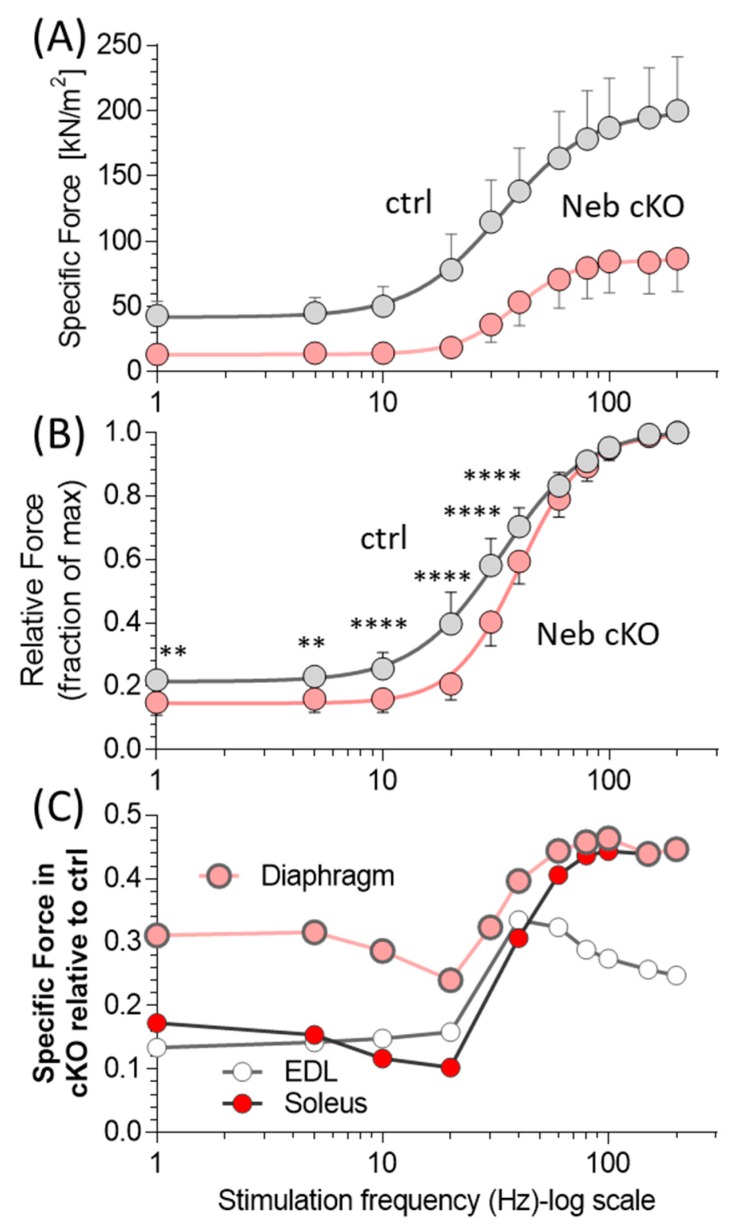
Force–frequency relations in control (ctrl) and of nebulin-based NM (Neb cKO) diaphragm muscle strips. (**A**) Force–frequency curves. Forces are shown as specific force (force normalized to the muscle’s cross-sectional area). Standard two-way ANOVA reveals that genotype significantly (*p* < 0.0001) affects specific force. A post-hoc test (Sidak) corrected for multiple comparisons reveals that at all stimulation frequencies, Neb cKO produces significantly less force than ctrl (two lowest frequencies *p* < 0.01, all other frequencies *p* < 0.0001). (**B**) Relative force–frequency curves. For each genotype, force is normalized to the maximal force at 200 Hz. The Neb cKO curve has a different shape than the ctrl curve. Standard two-way ANOVA reveals that genotype significantly (*p* < 0.0001) affects relative force. A post-hoc test (Sidak) corrected for multiple comparisons reveals that Neb cKO produces significantly less relative force than ctrl at 1–40 Hz (** *p* <0.01, **** *p* < 0.0001). (**C**) Force of Neb cKO muscles relative to that of ctrl diaphragm (this study) and EDL and soleus (data from Li et al. [13]), which uses the same mouse model (at 6 months old) and experimental protocols as the present study). Force normalized to the force of ctrl muscle at each frequency. Results from 15 ctrl and 18 Neb cKO mice. Mean ± SD are shown in A and B and only mean in C. Note that the x-axis uses a log-scale and that at many frequencies SD lines fall within the symbols.

At all stimulation frequencies, the specific force of diaphragm muscle strips was lower in Neb cKO mice than in ctrl mice (Figure 1A, Supplementary Table S1). The maximal tetanic force (stimulation frequency 200 Hz) of the cKO diaphragm was reduced by 57%, from 200 mN/mm^2^ to 86 mN/mm^2^ (*p* < 0.001) (Table 1). Note that in both genotypes there were only small increases in tetanic force at stimulation frequencies beyond 100 Hz. Dividing force at each stimulation frequency by the maximal tetanic force revealed relative force–frequency curves that were distinct in the two genotypes (Figure 1B), with the most severe force reduction in cKO diaphragm at stimulation frequencies <~30 Hz. The Hill-fits to the relative force–frequency relation showed in cKO diaphragm a significantly higher frequency needed to elicit half-maximal force (F_50_ frequency) 33 vs 38 Hz and a Hill coefficient that was significantly higher than in ctrl, 1.5 vs 2.5 (Table 1) for ctrl and Neb cKO, respectively. 

The force of the cKO relative to ctrl is shown as a function of frequency in Figure 1C. The force, relative to ctrl, is depressed most at frequencies ≲20 Hz and the difference becomes smaller at higher frequencies. This unique frequency-dependent force deficit behavior is also apparent in earlier results [13] obtained on intact extensor digitorum longus (EDL) and soleus muscle (results reproduced in Figure 1C). Thus in both diaphragm and peripheral muscles, nebulin-deficiency more severely affects force at low–intermediate stimulation frequencies. It is also noteworthy that comparison between diaphragm and peripheral muscle types (all intact muscles, stimulated electrically) show that the force deficit in cKO (relative to ctrl) was less severe in diaphragm than the peripheral muscles (Figure 1C). 

### 2.2. Effect of Tirasemtiv on Diaphragm Muscle Strip 

**Figure 2:**
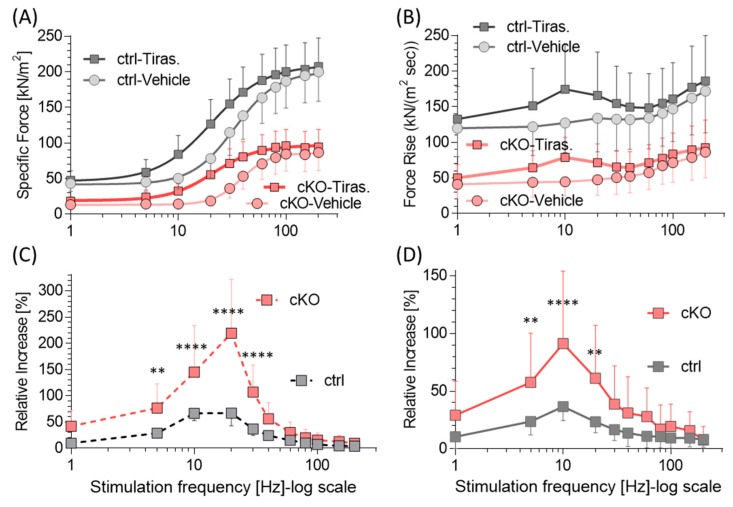
Effect of *tirasemtiv* on force production in control (ctrl) and Neb cKO diaphragm muscle strips. (**A** and **B**) Force–frequency curves (**A**) and maximal speed of specific force rise (dF/dt) (**B**) in ctrl and cKO muscle with vehicle or *tirasemtiv*. Two-way ANOVA with repeated measures reveals in both genotypes that *tirasemtiv* significantly (*p* < 0.0001) affects specific force. Post-hoc tests (Sidak) corrected for multiple comparisons reveal that *tirasemtiv* increases force at all stimulation frequencies except 1 Hz and maximal force rise at all stimulation frequencies, except 200 Hz for cKO (*p* < 0.01). (**C** and **D**) Effect of *tirasemtiv* on force (**C**) and speed of force rise (**D**) over vehicle at various stimulation frequencies. In both ctrl and cKO diaphragms, *tirasemtiv* increases force (**C**) and speed of force rise (**D**) with a maximal effect at ~20 Hz. A standard two-way ANOVA reveals that *tirasemtiv* has a significantly (*p* < 0.0001) different effect on force in Neb cKO diaphragm strips than in ctrl strips. Post-hoc tests (Sidak) corrected for multiple comparisons reveals that Neb cKO muscle has significantly greater enhancement with *tirasemtiv* than ctrl muscle in the mid-frequency range (** *p* < 0.01, **** *p* < 0.0001). (Results from 15 ctrl and 18 cKO mice. Mean ± SD are shown. Note that at several frequencies SD lines fall within the symbols.).

The effect of the FSTA *tirasemtiv* on specific force was evaluated in ctrl and NM diaphragm muscle. *Tirasemtiv* increased specific force, in both genotypes and at all stimulation frequencies (Figure 2A, Table 1, Suppl Table S1). Similarly, the maximal speed of specific force rise (dF/dt max) increased in both genotypes (Figure 2B). The increase in both specific force and speed of force rise was low at the lowest tested frequencies, increased sharply with frequency to reach a peak at ~20 Hz, and then fell again (Figure 2C,D). Although this general pattern was similar in the two genotypes, the average increase in specific force and speed of force rise were much higher in the cKO. In particular, the average specific force increase due to *tirasemtiv* across all frequencies was 72 ± 21% in cKO and 27 ± 7% in ctrl. The maximal difference between the genotypes peaked at ~20 Hz where *tirasemtiv* increased specific force by 219 ± 24% in cKO and 67 ± 6% in ctrl. 

**Figure 3:**
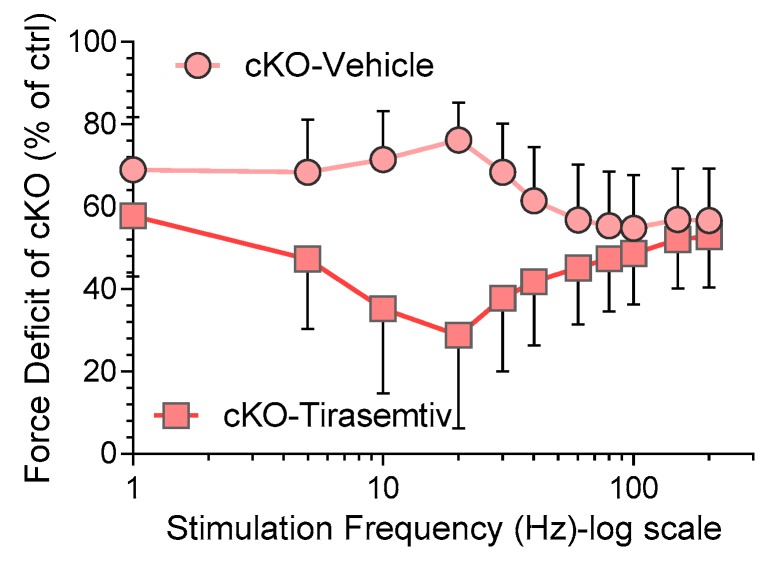
*Tirasemtiv* reduces the force deficit of cKO diaphragm strips. Specific force of cKO in vehicle (circles) or *tirasemtiv* (squares) relative to ctrl (in vehicle). A standard two-way ANOVA reveals that *tirasemtiv* has a significant (*p* < 0.0001) effect on the force deficit. Post-hoc tests (Sidak) corrected for multiple comparisons reveal that *tirasemtiv* significantly reduces the force deficit of cKO diaphragm at all stimulation frequencies up to 100 Hz (*p* < 0.001) with the largest effect at intermediate stimulation frequencies (e.g., at 20 Hz the deficit goes from 76% to 29%). Mean ± SD are shown.

The force deficit of the cKO diaphragm relative to ctrl (no *tiramsemtiv*) is shown in Figure 3. *Tirasemtiv* reduced the force deficit of cKO, averaged across all frequencies, from 62.5 ± 2.4% in absence of *tirasemtiv* to 45.0 ± 2.7% with *tirasemtiv*. The largest effect was found at 20 Hz where the deficit fell from 76% to 29%. Thus, *tirasemtiv* greatly improves isometric force production, especially at intermediate stimulation frequencies. 

**Figure 4:**
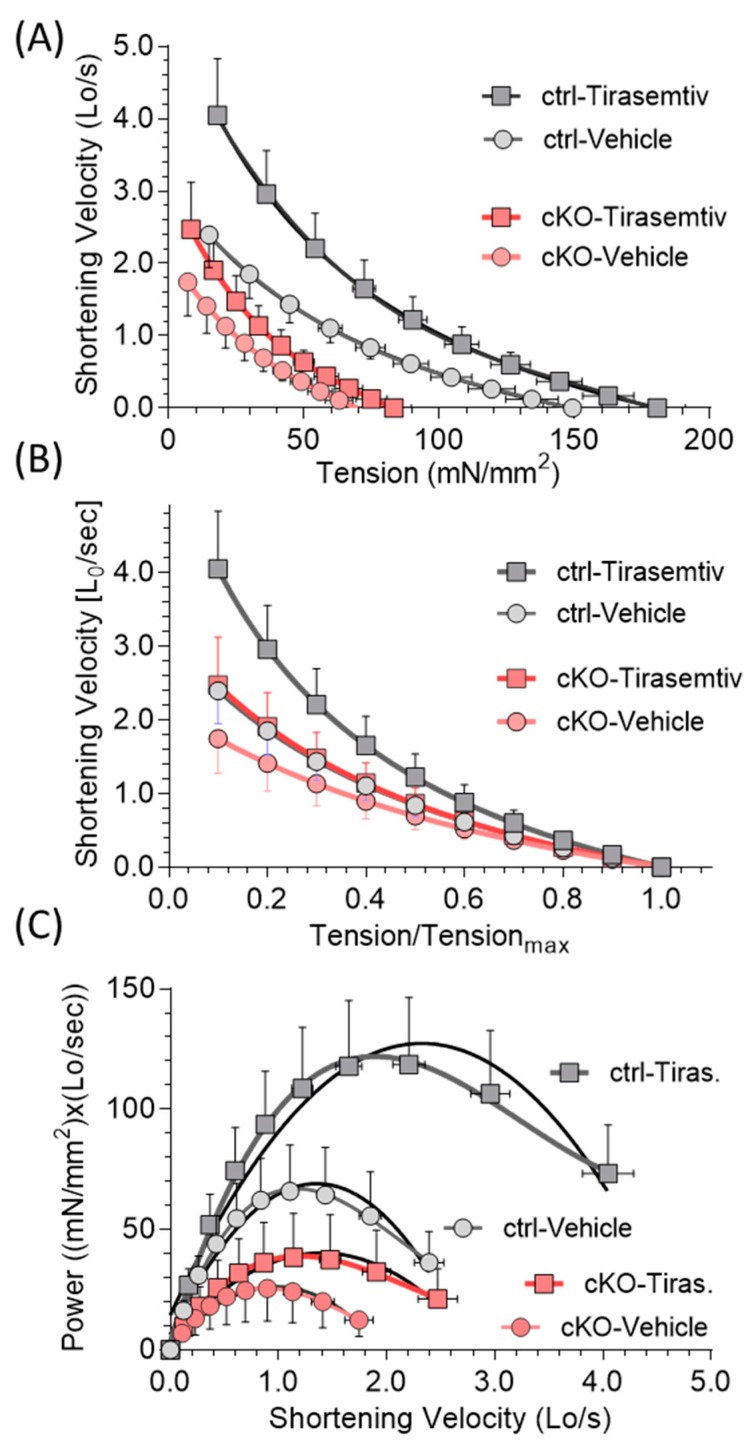
Effect of *tirasemtiv* on tension-velocity relation and power-velocity relations of diaphragm in control (ctrl) and Neb cKO mice. (**A**) Tension-velocity relationship measured in load-clamp experiments. At all load levels and in both genotypes, *tirasemtiv* significantly increases shortening velocity (*p* < 0.0001). (**B**) Tension-velocity relation with tension normalized to maximal tetanic tension. *Tirasemtiv* increases the shortening velocity at each tension level in both genotypes and statistical significance was found at Tension/Tension_max_ of 0.1–0.7 for ctrl and 0.1–0.6 for cKO (*p* < 0.01). Shortening velocity in cKO with tirasemtiv overlaps with that of ctrl in vehicle. (**C**) Power–velocity relationship. In both genotypes, maximal power is significantly increased by *tirasemtiv* (*p* < 0.0001) and maximal power occurs at higher shortening speeds. (Shortening speed expressed as Lo (length at optimal force)/second). Results from 11 ctrl mice and 13 cKO mice. Mean ± SD are shown. Note that at many frequencies SD lines fall within the symbols.

Load-clamp experiments were performed in which the diaphragm was tetanized at a 50 Hz stimulation frequency (this frequency was selected because it is the lowest stimulation frequency that results in a fused tetanus, avoiding velocity oscillations during the load-clamp period) and when force was maximal, the force was abruptly decreased and held constant at a preset value, allowing isotonic shortening speed to be measured and mechanical power to be calculated (as the product of force and velocity of shortening, see Methods for details). The shortening velocity of cKO diaphragm was much lower than that of ctrl diaphragm, both with force expressed on an absolute scale (Figure 4A) and with force normalized to the isometric force (Figure 4B). Power curves (Figure 4C) were much lower in cKO, as expected, with a reduction in maximal power of 62% in cKO relative to ctrl (Table 1). *Tirasemtiv* resulted in an increase of shortening velocity in both ctrl and cKO (Figure 4A,B). The *tirasemtiv*-based shortening velocity increase was larger in the ctrl (average at all load clamp levels: 48.9 ± 3.5%) than in the cKO diaphragm (average at all load clamp levels: 26.2 ± 2.8%). Importantly, the maximal power deficit (relative to ctrl in the presence of vehicle) was reduced from 62% without *tirasemtiv* to 42% with *tirasemtiv* (Table 1). Thus, *tirasemtiv* increases the shortening velocity in both genotype and reduces the power deficit of cKO diaphragm. 

### 2.3. Effect of Tirasemtiv on in Vivo Muscle Strength of Gastrocnemius Complex

**Figure 5:**
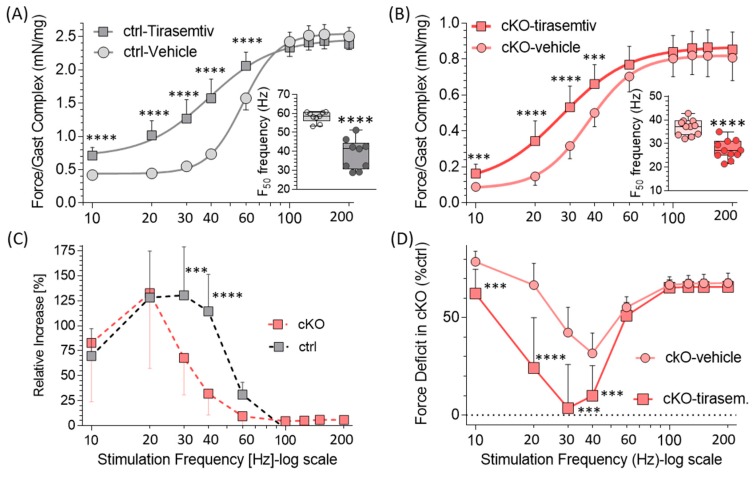
Force–frequency relation of in vivo gastrocnemius muscle complex measured in vehicle or tirasemtiv in ctrl and Neb cKO mice. (**A** and **B**) Force in *tirasemtiv* (square) and in vehicle (circle) in ctrl (A) and cKO (B) mice. Two-way ANOVA with repeated measures reveals in both genotypes that *tirasemtiv* significantly (*p* < 0.0001) affects force in ctrl (**A**) and cKO (**B**) mice. Post-hoc tests (Sidak) corrected for multiple comparisons reveal that *tirasemtiv* increases force at low–intermediate frequencies. Insets show frequency that results in half-maximal force, F_50_ frequency (light color: vehicle, dark color-*tirasemtiv*). An unpaired two-tailed t-test reveals in both genotypes a significant reduction in F50. (**C**) Effect of *tirasemtiv* on force over vehicle at various stimulation frequencies. In both ctrl and cKO, *tirasemtiv* increases force with a maximal effect at ~20 Hz. A standard two-way ANOVA with post-hoc tests (Sidak) corrected for multiple comparisons reveals that *tirasemtiv* has a significantly larger effect on ctrl gastrocnemius than cKO at 30 and 40 Hz (*** *p* < 0.001, **** *p* < 0.0001). (**D**) Force deficit of cKO gastrocnemius complex. Force of cKO in vehicle (circles) or *tirasemtiv* (squares) relative to ctrl (vehicle). A standard two-way ANOVA with Post-hoc tests (Sidak) corrected for multiple comparisons reveals that *tirasemtiv* reduces the force deficit of cKO gastrocnemius with a maximal reduction at intermediate stimulation frequencies (e.g., at 30 Hz the deficit goes from 42% to 4%). (Results from nine ctrl mice and 11 cKO mice. Mean ± SD are shown. Note that at many frequencies, SD lines fall within the symbols.).

A separate group of mice was used to test the effect of *tirasemtiv* on in vivo gastrocnemius muscle performance (see Methods). Specific force was lower in cKO muscle at all stimulation frequencies. The maximal tetanic force was 0.81 ± 0.04 mN/mg in Neb cKO vs. 2.50 ± 0.07 mN/mg in ctrl, i.e., a reduction of 68% in the cKO. *Tirasemtiv* left-shifted the force–frequency curves in both ctrl and Neb cKO mice, due to the large force increase at low–intermediate stimulation frequencies and the minimal effect at frequencies that give rise to (near) maximal tetanic force levels (Figure 5A,B). The frequency dependence of the force increase is shown in Figure 5C. The average force increase at stimulation frequencies of 10–60 Hz was 95 ± 19% in ctrl and 65 ± 21% in cKO mice. Because of the frequency-dependent *tirasemtiv* effect, the frequency for half-maximal tension (F_50_ frequency) was significantly reduced by *tirasemtiv*, in both genotypes from 58 to 39 Hz in ctrl and 37 to 27 Hz in Neb cKO (Table 1 and Figure 5A,B insets). 

The effect of *tirasemtiv* on the force deficit of the cKO gastrocnemius complex relative to ctrl (no *tirasemtiv*) is shown in Figure 5D. *Tirasemtiv* reduced the force deficit from 55 ± 6% in absence of *tirasemtiv* to 30 ± 11% with tirasemtiv, averaged across 10–60 Hz. The largest *tirasemtiv* effect was found at 30 Hz where the deficit fell from 42% to 4%. Thus, at low–intermediate frequencies, *tirasemtiv* greatly improves the in vivo strength of the gastrocnemius complex. 

## 3. Discussion

Respiratory failure due to diaphragm dysfunction is considered a main cause of death in NM [2], necessitating a need to understand how NM affects diaphragm function. Here we studied both isometric force and isotonic shortening velocity of diaphragm muscle strips in a mouse model of nebulin-based NM (Neb cKO). A large contractile deficit was found in nebulin-deficient intact muscle that is frequency dependent, with the largest deficits at low–intermediate stimulation frequencies. The effect of the troponin activator *tirasemtiv* as a possible therapeutic was examined. *Tirasemtiv* had no effect at maximal stimulation frequencies, but greatly reduced the force deficit of cKO diaphragm at sub-maximal activation. Similar effects were found in the gastrocnemius muscle complex. Below we discuss these findings in detail. 

### 3.1. Contractile Performance in Neb cKO Diaphragm

Nebulin deficiency results in a large force deficit in the diaphragm strips, between ~70% (low stimulation frequencies) and ~55% (high stimulation frequency). This frequency dependence can be explained by the reduced calcium sensitivity of force generation (increased calcium level required to reach half-maximal force) that has been found in nebulin-deficient permeabilized muscle fibers [21,29]. This is expected to reduce force in intact cKO muscle at submaximal stimulation frequencies. Because the reduction in force of permeabilized fibers in cKO only occurs at submaximal activation levels, altered calcium sensitivity is unlikely to play a role in intact muscle that is maximally stimulated (200 Hz). The large reduction in maximal tetanic force (~55%) is likely due to other factors including a reduced thin filament length [13,20,37], a reduced number of force generating cross-bridges [13,24,26,27,38,39], and less force per attached cross-bridge [40]. Changes in cross-bridge cycling are also evident from the lower shortening velocity that was found in this study (Figure 4). Some of these changes could in principle be due to fiber type switching but unlike in other studied muscle types of Neb cKO mice, fiber type switching in diaphragm muscle is minimal [13] and is unlikely to play a major role in our diaphragm study. In summary, a large force deficit occurs in electrically stimulated diaphragm strips of Neb cKO mice that can be explained by structural and functional changes in the myofilaments with a frequency dependence that is likely due to reduced calcium sensitivity at submaximal activation levels. 

Previous studies on biopsies of NM patients indicated that the diaphragm is more affected than peripheral skeletal muscles [41], a conclusion based on the presence of more nemaline rods in the diaphragm than in non-respiratory muscles. Earlier functional studies on permeabilized fibers from a nebulin-deficient mouse model (Neb^ΔExon55^ model) tested this notion [29]. Maximally activated fibers from the diaphragm had a higher force deficit than soleus muscle fibers [29], lending support to the view that the diaphragm is unusually sensitive to NM-causing mutations. However, our present study on intact muscle reveals that the force deficit of the diaphragm is less than that of the EDL and soleus muscle (Figure 1C), particularly at submaximal stimulation frequencies, and less than in the gastrocnemius, as measured in vivo (Figure 5). The explanation for the differences in results between the present and earlier study [29] is unclear but might involve the more severe pathology in Neb^ΔExon55^ mice (mice die within the first week of life) and the milder pathology in the Neb cKO model, with survival into adulthood. Although the diaphragm might not be more sensitive to dysfunction than peripheral muscles, the severe contractility deficit of the diaphragm is a likely major cause of weakness in NM patients. The diaphragm is the main muscle of inspiration, taking up some seventy percent of the work of breathing in humans [29] and, furthermore, the diaphragm is highly active with a duty cycle (ratio of active to inactive time) of ~35% [42], several-fold higher than for peripheral muscles [43]. Thus, obtaining therapeutic approaches that increase diaphragm strength is critically important.

### 3.2. Tirasemtiv Effect on Diaphragm

*Tirasemtiv* is a small molecule that selectively activates fast skeletal muscle by slowing the rate of calcium release from the troponin complex [30,44]. It increases the stability of the open conformation of the fast skeletal muscle troponin/tropomyosin complex and enhances cross-bridge formation at a given submaximal calcium concentration [44]. *Tirasemtiv* has been investigated as a potential therapy for neuromuscular disorders, such as Amyotrophic Lateral Sclerosis (ALS) and, myasthenia gravis [31,32,33,34,44]. CK-2066260, another FSTA [22,35] from a similar chemical class as *tirasemtiv*, has also been studied in permeabilized fibers activated with exogenous calcium, using fibers from the Neb^ΔExon55^ mouse model [36] and from NM patients [36]. In those studies, calcium sensitivity was significantly increased by *tirasemtiv* as reflected by the increased submaximal force levels. Here we studied the effect of *tirasemtiv* on electrically stimulated ctrl and Neb cKO diaphragm muscle. *Tirasemtiv* enhanced the specific force of diaphragm muscle strips in both genotypes. This effect reached a maximum at ~20 Hz (219% ± 24%) and then gradually decreased as stimulation frequencies were increased (Figure 2C). *Tirasemtiv* also increased the maximal speed of force rise (dF/dt max) in both genotypes with a similar frequency dependence to that of specific force (Figure 2B,D); increasing both the speed of force rise and the final force level are likely to positively impact breathing function [45]. Although *tirasemtiv* affects both the ctrl and Neb cKO diaphragm, the magnitudes of the effects are much greater in nebulin-deficient muscle than in ctrl muscle (Figure 2C,D). This might be explained by the reduced calcium sensitivity of Neb cKO muscle, relative to ctrl, which results in a lower fraction of attached cross-bridges during submaximal activation [21,24,26,27], providing more ‘range’ for additional activation by *tirasemtiv*. In summary, *tirasemtiv* greatly improves the contractility of the diaphragm and the larger effect in Neb cKO diaphragm is fortuitous. 

### 3.3. Contractility of Gastrocnemius Complex and Effects of Tirasemtiv

The in vivo contractility of the gastrocnemius complex was severely depressed in the Neb cKO mouse with a maximal specific force that was reduced by 68% (Table 1). The frequency for half-maximal force (F_50_ frequency) was reduced in the gastrocnemius (from 58 Hz in ctrl to 37 Hz in Neb cKO), in contrast to the diaphragm where an increase was found (from 33 Hz to 38 Hz). This reduction in F_50_ frequency of the gastrocnemius might reflect the fiber-type switch that occurs in this muscle from <5% type-I in ctrl mice to ~40% type-I in Neb cKO and the lower F_50_ frequency of type I fibers, compared to type II fibers [13]. This lower F_50_ frequency of type I fibers is likely due to their higher calcium sensitivity [46,47]. *Tirasemtiv* significantly increased the contractile force of the gastrocnemius complex in both genotypes at stimulation frequencies less than 60 Hz (Figure 5C). However, unlike in the diaphragm, the effect was not larger in the Neb cKO muscle relative to ctrl. As discussed above, the increased sensitivity of the Neb cKO diaphragm to *tirasemtiv* is likely due to the reduced myofilament calcium sensitivity of this muscle type, but this effect might be negated in the gastrocnemius muscle complex due to fiber type switching away from type II to type I fibers (where *tirasemtiv* does not have an effect). Regardless, there is a robust effect of *tirasemtiv* on muscular strength of the gastrocnemius complex resulting in a much reduced force deficit in Neb cKO mice (Figure 5D). Considering that skeletal muscles, including diaphragm muscle, operate at submaximal activation levels during normal activity (between 10% and 65% of its maximal capacity) [47,48,49], the beneficial effect of *tirasemtiv* at submaximal activation levels suggest that *tirasemtiv* might be useful for treatment of nebulin-based NM patients. 

### 3.4. Conclusion

Intact muscles from Neb cKO mice stimulated either electrically (diaphragm) or via its innervating nerve (gastrocnemius complex) produce, relative to ctrl muscles, much reduced levels of force. FSTA *tirasemtiv* robustly enhances contractility in sub-maximally stimulated intact muscles and has therefore therapeutic potential in diaphragm as well as in peripheral muscle. Whether these findings in the mouse can be extrapolated to other species, including human, will depend on whether fast fibers (type II fibers) are expressed at relatively high levels. Slow fibers (type I fibers) and fast fibers are present in approximately equal proportion in the adult human diaphragm while intercostal muscles contain a higher proportion of fast fibers [50]. Peripheral muscle in humans contain between 25% and 50% type II fibers [51]. Thus, the fiber type composition of human muscles is favorable for a significant *tirasemtiv* effect. An additional requirement for *tirasemtiv* to work is a physiological activation level that is submaximal. It has been previously shown that during normal breathing, the diaphragm muscle is activated far below its maximal activation level and generates ~25% of its maximal force [45,52] and evidence exists that peripheral muscle are also submaximally activated during normal daily activities [49]. Thus, *tirasemtiv* and other FSTAs are likely to augment breathing as well as peripheral muscle function in mouse and human. *Reldesemtiv,* a next generation FSTA, is currently under clinical investigation in ALS and spinal muscular atrophy, studies that include measures of respiratory and peripheral muscle function. Overall, fast skeletal muscle troponin activators might also be suitable for treating diaphragm dysfunction in patients with nebulin-based NM, where reduced myofilament calcium sensitivity is a hallmark characteristic of the underlying muscle dysfunction. 

## 4. Material and Methods

### 4.1. Animal Model

The conditional nebulin knockout (Neb cKO) mouse that was used has previously been described [13]. All mice are on a C57BL/6J background. Floxed mice were bred to a MCK-Cre strain that expresses Cre recombinase under control of the Muscle Creatine Kinase (MCK) promoter. Mice that were positive for MCK-Cre and homozygous for the floxed nebulin allele were nebulin deficient and are referred to as Neb cKO. Mice with at least one nebulin WT allele and being either MCK-Cre positive or negative were grouped as control (ctrl) mice. The experiments were approved by the University of Arizona Institutional Animal Care and Use Committee (Project number 09-057) and were in accordance with the United States Public Health Service’s Policy on Humane Care and Use of Laboratory Animals. 

### 4.2. Tirasemtiv

*Tirasemtiv* was provided by Cytokinetics (South San Francisco, CA, USA). For the ex-vivo diaphragm study, *tirasemtiv* was dissolved in dimethylsulfoxide (DMSO) to make a stock solution, and then it was added directly to the experimental solution to prepare the final desired concentration of *tirasemtiv* (3 μM) in 1% DMSO. The concentration of *tirasemtiv* (3 μM) was chosen based on previous studies that showed that this concentration effectively increases sub-maximal force in skeletal muscle [31,32]. In the in vivo studies to measure in vivo function of the gastrocnemius complex, a single dose (10 mg/kg) of *tirasemtiv* dissolved in 10% DMA, 50% PEG400 and a 40% Cavitron cyclodextrin formulation at a concentration of 3 mg/mL was delivered via intraperitoneal (IP) injection. A separate control group received vehicle injections for comparison.

### 4.3. Ex-Vivo Diaphragm Muscle Mechanics

#### 4.3.1. Muscle Experiments

Four month old Neb cKO (136 ± 2 days old) and control (ctrl) (137 ± 1 days old) mice were used for this study. Mice were anesthetized with isoflurane and sacrificed by cervical dislocation. Immediately, the whole diaphragm muscle was excised and a small muscle strip from central tendon to ribcage on the left side of diaphragm was isolated with parallel fiber alignment. Muscle strips were mounted to the isolated muscle test system (1200A, Aurora Scientific Inc., Aurora, ON, Canada) that has been described previously [53]. In brief, tendon and floating rib were tied with 7-0 silk sutures, and the muscle was attached between the lever arm of dual servomotor-force transducer (300C, Aurora scientific, Aurora, ON, Canada) and a fixed hook. The experimental bath was filled with Krebs-Ringer solution containing (in mM) 137 NaCl, 5 KCl, 1 MgSO_4_, 2 CaCl_2_, 1 NaH_2_PO_4_, 24 NaHCO_3_, 11 Glucose with 95%/5% O_2/_CO_2_ supply (pH = 7.4). The temperature during the experiments was controlled at 30 °C. The muscle was placed between platinum electrodes connected to an electrical stimulator (701C, Aurora scientific, Aurora, ON, Canada) for muscle activation. The optimal muscle length (L_0_) was determined by adjusting overall muscle length until maximal force was generated with a 400 ms 20 Hz stimulation (pulse duration of 200 μs with biphasic polarity). The muscle length at L_0_ was measured using a digital caliper. At L_0_, an initial tetanic contraction at 150 Hz stimulation frequency was imposed to evaluate the quality of the muscle preparation. Measured forces were normalized to the cross-sectional area (CSA) to obtain specific force (mN/mm^2^). CSA (in mm^2^) was calculated using muscle mass (in g), fiber length (in mm), and muscle density (1.056 g/mm^3^); CSA = muscle mass / (muscle length × muscle density).

#### 4.3.2. Protocols

##### Force–Frequency Protocol

To establish the force–frequency relation, active forces at various stimulation frequencies were measured. 15 ctrl and 18 Neb cKO mice were used for the force–frequency protocol. The muscle was stimulated at 1, 5, 10, 20, 30, 40, 60, 100, 150, and 200Hz and waiting for 30, 30, 60, 90, 90, 120, 120, 180, 180, and 180 s, respectively, in between each stimulation. The durations of stimulation were 0.4 s for 1–40 Hz and 0.8 s for 60–200 Hz of stimulation. The first force–frequency protocol was performed in the vehicle solution, and the experimental solution was then switched to *tirasemtiv* solution. *Tirasemtiv* was dissolved in dimethylsulfoxide (DMSO) and directed added to the experimental solution to prepare desired final concentration (3 μM). The muscle was incubated in *tirasemtiv* solution for 10 min with 30 Hz stimulation frequency every minute to monitor changes in force. When the increase in force was stable, the force–frequency protocol in *tirasemtiv* solution was measured. In order to limit the chance on a false negative result due to the incomplete removal of *tirasemtiv*, we consistently performed the vehicle condition first and then the *tirasemtiv* condition. 

##### Force–Velocity Protocol

To determine the force-velocity relation, load-clamp experiments were performed. Shortening velocities were measured during isotonic contraction against loads 85%, 70%, 55%, 40%, 25%, and 10% of maximal force at 50 Hz of stimulation frequency. Each contraction started out with a 200 ms isometric phase (to reach the plateau force), and then a 150 ms load-clamp was applied. A series of load-clamps was performed in solution with vehicle and the same protocol was then repeated in *tirasemtiv* solution. Before the protocol was repeated with *tirasemtiv*, the muscle was contracted with a 30 Hz stimulation frequency (0.4 s in duration) every minute until tension stopped to increase. After completion of each experiment, the sutures and tendons were carefully removed, and the muscle mass was then measured. 

##### Analysis

The force–frequency curve was fit with a Hill equation to calculate the frequency that results in 50% of maximal force (F_50_ frequency) and the Hill coefficient (n_H_), an index of the steepness of the curve. The maximal speed of specific force rise was analyzed as the maximum of the derivative of force with respect to time (dF/dt) during the force rise phase. Shortening velocity (in mm/sec) was measured as a slope of linear portion of the shortening period (~40 ms duration) after force was stable. For each preparation, shortening velocity was normalized by muscle fiber length. Muscle power production was calculated by multiplying the shortening velocity and the applied load. Force–power curves were fitted by 2nd order polynomial non-linear regression, and the maximal power production was determined from the apex of the force–power curve.

### 4.4. In Vivo Strength Measurement of Gastrocnemius Muscle Complex 

Two month old Neb cKO (54 ± 2 days old) and ctrl (53 ± 2 days old) mice were tested using an in vivo muscle analysis apparatus (model 1300A; Aurora scientific, Aurora, ON, Canada). In order to test the effect of *tirasemtiv* on the in vivo muscle strength, we selected the gastrocnemius muscle complex due to the large population of fast skeletal muscle fibers and the convenience of in situ force measurement. Subcutaneous needle electrodes were positioned along the sciatic nerve, distal to the peroneal nerve to induce plantar flexion. The minimum current required to induce maximal force was used to avoid stimulating antagonist muscle groups. Contractile force of the gastrocnemius muscle complex was measured through the movement of the foot that was attached to the footplate force measurement system. Mice were first sedated under anesthesia using 1–2% of isoflurane with O_2_ supply and placed on the experiment platform. Body temperature was maintained with a circulated water bath system. Hair around the experimental area of the right leg was removed with an electric razor. The leg was immobilized using a blunt knee clamp and the ankle joint positioned at a 90° angle with the foot secured to the force transducer footplate using adhesive tape. Two needle electrodes were placed distal to the peroneal nerve to stimulate the gastrocnemius muscle complex. The optimum pulse phase polarity was determined using a 10 Hz stimulation frequency at 3mA and the current set using a series of twitches repeated every 10 s until maximum forces were obtained. Separate groups of mice were used to test the effect of *tirasemtiv* on in vivo strength of the gastrocnemius complex. The treatment group received a single dose of *tirasemtiv* (10 mg/kg dissolved in 50% PEG400: 40% (40% g/mL Cavitron in water): 10% DMA) acutely via intraperitoneal (IP) injection, while a vehicle solution (same carrier as used for *tirasemtiv*) was administered to the control group for comparison. Pre- and post- treatment tests were also conducted to serve as internal controls. Following the procedure, animals were euthanized and the gastrocnemius complex (soleus, plantaris and gastrocnemius) were dissected and weighed for the normalization of measured force (mN/mg). 

#### Protocols

To establish the force–frequency relation, gastrocnemius complex muscle forces at various stimulation frequencies were measured. 9 ctrl and 11 Neb cKO mice were used for the force–frequency protocol in both the treatment and vehicle groups. The gastrocnemius complex was stimulated at 10, 20, 30, 40, 60, 100, 125, 150, and 200 Hz, waiting for 30, 30, 30, 60, 60, 120, 120, and 120 s, respectively, in between each stimulation. A pulse width of 200 µs and 350 ms duration was used for all tetanic stimulations. The first force–frequency protocol was performed under baseline conditions. Following a brief rest period, each mouse received an IP injection containing either a 10 mg/kg dose of *tirasemtiv* or vehicle only. A 30 Hz stimulation frequency was repeated every minute for a minimum of 10 min to monitor changes in force. When the increase in force was stable, the force–frequency protocol was repeated. The force–frequency curve was fit with a Hill equation to calculate the F_50_ frequency and the Hill coefficient (nH), an index of the steepness of the curve. 

### 4.5. Statistical Analysis 

Data are presented as mean ± SD. We used a repeated measure two-way ANOVA with post-hoc tests using the Sidak method with multiple comparisons correction, unless indicated otherwise. In order to identify the effect of *tirasemtiv* at each genotype, the two factors for analysis were frequency and treatment (vehicle or *tirasemtiv*; Figure 2A,B, Figure 3, Figure 4B, Figure 5A,B,D). For comparison of genotype, the two factors for analysis were frequency and genotype (ctrl vs. cKO; Figure 1A,B, Figure 2C,D, Figure 5C). *p* < 0.05 was considered to be statistically significant. GraphPad Prism 6 was used for statistical analysis.

## Figures and Tables

**Table 1:** Effect of *tirasemtiv* on muscle performance of ctrl and Neb cKO mice.

	Ctrl		cKO	
	Vehicle	*Tirasemtiv*	Vehicle	*Tirasemtiv*
Diaphragm	*n* = 15	*n* = 15	*n* = 18	*n* = 18
Specific force (mN/mm^2^) at 200 Hz	200.0 ± 12.0	207.6 ± 11.7***	86.8 ± 7.0####	94.4 ± 6.9***,####
F_50_ frequency (Hz)	33.4 ± 2.7	20.3 ± 2.3****	38.3 ± 2.4####	21.1 ± 2.1****
nH	1.47 ± 0.1	1.05 ± 0.05	2.49 ± 0.13####	1.38 ± 0.09****
Velocity @ 0.1Fmax(Lo/sec)	2.39 ± 0.1	4.05 ± 0.2****	1.75 ± 0.13####	2.47 ± 0.18****
Max Power((mN/mm^2^)x(Lo/s))	65.0 ± 5.4	115.7 ± 7.9****	24.6 ± 3.7####	37.5 ± 5.3****
Gastrocnemius complex	*n* = 9	*n* = 9	*n* = 11	*n* = 11
Specific force (mN/mg) at 200 Hz	2.50 ± 0.07	2.39 ± 0.08	0.81 ± 0.04####	0.85 ± 0.03####
F_50_ frequency (Hz)	57.9 ± 1.3	39.1 ± 2.7****	37.2 ± 1.4####	27.3 ± 1.5**#
nH	4.9 ± 0.5	2.7 ± 0.5**	3.6 ± 0.5#	2.5 ± 0.5*

Asterisk: significant difference between *tirasemtiv* and vehicle; hashtag: significant difference between genotypes.

## Supplementary Materials

Supplementary materials can be found at https://www.mdpi.com/1422-0067/20/20/5008/s1.
