# Supplementary material for: Functional Characterization of the Intact Diaphragm in a Nebulin-Based Nemaline Myopathy (NM) Model-Effects of the Fast Skeletal Muscle Troponin Activator tirasemtiv
Source: Int J Mol Sci. 2019 Oct 10;20(20):5008. doi: 10.3390/ijms20205008 (PMC6829460; doi:10.3390/ijms20205008)
Supplement: Supplementary file 1 [file ijms-20-05008-s001.pdf]

Supplementary Table S1. Specific force in mN/mm² (force normalized to the muscle's cross-sectional area).

Stimulation Frequency (Hz)	**Ctrl (n = 15)**				**Neb cKO (n = 18)**			
	**Vehicle**		**Tirasemtiv**		**Vehicle**		**Tirasemtiv**	
	mean	SD	mean	SD	mean	SD	mean	SD
1	43.26	10.90	47.73	13.21	13.41	5.56	18.26	6.39
5	45.31	11.77	58.94	17.89	14.33	5.82	23.89	7.72
10	50.33	14.99	84.11	26.73	14.36	5.91	32.59	10.32
20	78.26	27.35	126.85	34.37	18.59	7.12	55.70	17.67
30	114.80	32.20	154.85	35.32	36.29	13.63	71.38	20.49
40	138.57	33.23	171.33	35.50	53.44	18.22	80.63	21.52
60	163.69	36.05	188.05	36.82	70.74	21.91	89.76	22.49
80	178.50	37.00	196.17	37.47	79.70	23.50	93.99	22.93
100	187.03	37.82	200.25	37.62	84.57	24.17	96.01	23.08
150	194.84	38.47	203.50	37.63	83.98	24.17	93.51	23.26
200	200.04	41.42	207.56	40.39	86.80	25.19	94.36	24.85
